# Single-cell analysis reveals congruence between kidney organoids and human fetal kidney

**DOI:** 10.1186/s13073-019-0615-0

**Published:** 2019-01-23

**Authors:** Alexander N. Combes, Luke Zappia, Pei Xuan Er, Alicia Oshlack, Melissa H. Little

**Affiliations:** 1 Department of Anatomy & Neuroscience, University of Melbourne, Melbourne, VIC Australia; 2 Murdoch Children’s Research Institute, Melbourne, VIC Australia; 3 School of Biosciences, The University of Melbourne, Melbourne, VIC Australia; 4 Department of Paediatrics, The University of Melbourne, Melbourne, VIC Australia

**Keywords:** Single-cell RNA sequencing, Human kidney organoids, Stem cell-derived models, Induced pluripotent cells, Organoids

## Abstract

**Background:**

Human kidney organoids hold promise for studying development, disease modelling and drug screening. However, the utility of stem cell-derived kidney tissues will depend on how faithfully these replicate normal fetal development at the level of cellular identity and complexity.

**Methods:**

Here, we present an integrated analysis of single cell datasets from human kidney organoids and human fetal kidney to assess similarities and differences between the component cell types.

**Results:**

Clusters in the combined dataset contained cells from both organoid and fetal kidney with transcriptional congruence for key stromal, endothelial and nephron cell type-specific markers. Organoid enriched neural, glial and muscle progenitor populations were also evident. Major transcriptional differences between organoid and human tissue were likely related to technical artefacts. Cell type-specific comparisons revealed differences in stromal, endothelial and nephron progenitor cell types including expression of WNT2B in the human fetal kidney stroma.

**Conclusions:**

This study supports the fidelity of kidney organoids as models of the developing kidney and affirms their potential in disease modelling and drug screening.

**Electronic supplementary material:**

The online version of this article (10.1186/s13073-019-0615-0) contains supplementary material, which is available to authorized users.

## Background

Knowledge of developmental programs can be used to direct the differentiation of human-induced pluripotent stem cells towards a desired cell fate. Such approaches have successfully generated models of human intestinal epithelium, brain and ear, in each instance forming multicellular self-organising structures termed organoids by mimicking conditions that regulate development of the same tissues during embryogenesis [1]. Similarly, protocols for the generation of human kidney cell types have been developed by ourselves [2, 3] and others [4–8]. Such protocols raise the exciting prospect of disease modelling, toxicity and drug screening in vitro and open new opportunities for regenerative medicine. However, the value of stem cell-derived kidney tissue will depend on how faithfully it represents human renal tissue, the degree to which the component cell types mature and the absence of confounding cell types within such cultures.

Component cell types present within kidney organoids have primarily been defined by detecting established markers of murine renal cell types by immunofluorescence. This has identified cell types with similarity to endothelial cells (CD31^+^), stroma (MEIS1^+^), nephron progenitor cells (SIX2^+^, HOXD11^+^, WT1^+^, PAX2^+^), and epithelial structures with markers of the ureteric epithelium (PAX2^+^, GATA3^+^, CDH1^+^), renal vesicle (JAG1^+^), distal tubule (CDH1^+^, GATA3^−^), loop of Henle (UMOD^+^, CDH1^+^), proximal tubule (LTL^+^CDH1^−^, CUBN^+^) and podocytes (NPHS1^+^) [2, 3]. However, the extent to which cell identity is conserved beyond these key markers is unclear. Indeed, as these markers were selected based on an understanding of mouse kidney development, they are not definitive evidence of an appropriate human cell type. Similarly, there can be variation between kidney organoid experiments and differentiation protocols as well as between starting cell lines. Finally, it is likely that the differentiation is imperfect, resulting in variable populations of off-target cell types.

We recently profiled gene expression in over 8000 single cells from two batches of human pluripotent stem cell-derived kidney organoids as part of an analysis of experimental variation in our organoid protocol [9]. Here, we independently analyse that data to characterise the cellular composition of kidney organoids, extending the expression profiles of expected endothelial and nephron cell types, and identifying subpopulations expressing stromal markers, and off-target glial, neural and muscle progenitors. Despite expression of recognisable renal markers, there remains the possibility of broader underlying differences between kidney organoid cell types and equivalent populations in the developing human kidney. To directly compare these tissues, we integrated single-cell RNA sequencing (scRNA-seq) data from kidney organoids with publicly available human fetal kidney (hFK) data [10]. Our analysis shows that clusters corresponding to stromal, nephron and endothelial populations included cells from both organoid and hFK origin. We further identified populations that were specific to each dataset. Substantial conservation of cell type-specific markers between cells from hFK and organoids was observed whereas differences between tissues were obscured by a strong signature that was found to be technical rather than biological. After identifying and removing genes associated with that signature, cell type-specific differences in stromal, endothelial and nephron progenitor cells emerged, indicating avenues for improving organoid cell types. This analysis extends our understanding of the cellular composition of human kidney organoids and demonstrates that kidney organoids reproduce several cell types found in the developing human kidney.

## Methods

This study aimed to characterise the cellular composition of human kidney organoids and compare organoid cell types to equivalent cell types in the developing human kidney. Two batches of organoids were produced and profiled. The resulting scRNA-seq dataset was then integrated with and compared to publicly available human fetal kidney scRNA-seq data [10] as outlined below.

### Organoid differentiation and single-cell experiments

Kidney organoids were made and stained according to our published protocol [11] from human-induced pluripotent stem cell line CRL1502 [12]. Three organoid samples were differentiated to day 25 (7 days of monolayer culture plus 18 days as a 3D aggregate). A further organoid was differentiated to day 25 in a second independent experiment. Organoids were dissociated and run on 10x Chromium Single Cell Chips as previously described [9]. Additional organoids were differentiated to day 24 for validation of glial and muscle progenitor populations. Immunofluorescence was performed according to our published protocol using antibodies detailed in that report [11]. Additional antibodies to FABP7 (Abcam Rabbit anti-BLBP #ab32423) and MYOG (Abcam Mouse anti MYOG #ab1835) were used at 1:300.

### Pre-processing

The Cell Ranger pipeline (v1.3.1 10X Genomics) was used to perform sample demultiplexing, barcode processing and single-cell gene counting. Briefly, samples were demultiplexed to produce a pair of FASTQ files for each sample. Reads containing sequence information were aligned to the GRCh38 reference genome provided with Cell Ranger (v1.2.0). Cell barcodes were filtered to remove empty droplets, and PCR duplicates were removed by selecting unique combinations of cell barcodes, unique molecular identifiers and gene ids with the final results being a gene expression matrix that was used for further analysis. The three samples in the first batch of organoids were aggregated using Cell Ranger with no normalisation and treated as a single dataset.

### Quality control

The R statistical programming language (v3.5.0) [13] was used for further analysis. Count data for each experiment was read into R and used to construct a SingleCellExperiment object (v1.2.0) [14]. Gene annotation information was added from BioMart [15] using the biomaRt package (v2.36.1) [16], and cells were assigned cell cycle scores using the cyclone [17] function in the scran package (v1.8.2) [18].

The scater package (v1.8.2) [19] was used to produce a series of diagnostic quality control plots. Cells with a high number of expressed genes (indicating potential doublets) were removed, as were cells with a high percentage of counts assigned to mitochondrial or ribosomal genes, or with low expression of the housekeeping genes GAPDH and ACTB.

Genes that had less than two total counts across a dataset, or were expressed in less than two individual cells, were removed. We also removed genes without an annotated HGNC symbol.

Following quality control, the first organoid dataset consisted of 6649 cells and 18,386 genes with a median of 2738 genes expressed per a cell, the fourth organoid had 1288 cells and 16,885 genes with a median of 3248 genes expressed per cell and the human developing kidney dataset [10] had 3178 cells and 16,166 genes with a median of 1509.5 genes expressed per cell.

### Clustering analysis

#### Organoids

The two organoid datasets were integrated using the alignment method in the Seurat package (v2.3.1) [20, 21]. Briefly, highly variable genes were identified in each dataset and those that were present in both datasets (1156 genes) were selected. Canonical correlation analysis [22, 23] was then performed using the selected genes and 25 dimensions that represent the majority of variation were selected. The final step used dynamic time warping [24] to align the datasets in the selected subspace.

To perform clustering, Seurat constructs a shared nearest neighbour graph of cells in the aligned subspace and uses the Louvain modularity optimisation [25] to assign cells to clusters. The number of clusters produced using this method is controlled by a resolution parameter with higher values giving more clusters. We performed clustering over a range of resolutions from 0 to 1 in steps of 0.1 and used the Clustree package (v0.2.2.9000) to produce clustering trees [26] showing the expression of known marker genes to select the appropriate resolution to use. We chose to use a resolution of 0.6 which produced 13 clusters.

Marker genes for each cluster were detected by testing for differential expression between cells in one cluster and all other cells using a Wilcoxon rank sum test [27]. To reduce processing time, only genes that were expressed in at least 10% of cells in one of these groups were tested. We chose the 10% cutoff over the default of 25% in order to return results for more genes. To identify conserved marker genes, a similar process was performed on each dataset separately and the results combined using the maximum *p* value method. We also tested for within cluster differential expression to identify differences between cells of the same type in different datasets.

Based on identified marker genes, we determined clusters 2 and 9 represented the nephron lineage. The 1125 cells in these clusters were re-clustered at a resolution of 0.5 resulting in 5 clusters.

We also performed pseudotime trajectory analysis on the nephron cells using Monocle (v2.8.0) [28, 29]. The intersection of the top 100 genes with the greatest absolute fold change for each nephron cluster was selected for this analysis, giving a set of 455 genes used to order the cells.

#### Combined

The combined organoid and human fetal kidney analysis used the procedure described for the organoid-only analysis but with slightly different parameters. We identified 1368 variable genes present in all three datasets and selected the first 20 canonical correlation dimensions. For clustering, we chose a resolution of 0.5 which produced 16 clusters. Clusters 6, 7, 10 and 15 were determined to be the nephron lineage and these 1964 cells were re-clustered at a resolution of 0.6 producing 8 clusters.

We also performed differential expression testing between the two datasets as a whole, which was used to identify a signature of 374 genes that represent the main differences between them. To identify cell type-specific differences between organoid and human fetal kidney, we performed differential expression testing between cells within a cluster and removed genes found in the overall differential expression signature. Cluster 7 in the combined nephron analysis was identified as a human fetal kidney specific podocyte cluster. To investigate the differences between these cells and other podocytes, we compared gene expression in this cluster to the general podocyte cluster (CN0).

#### Visualisation and presentation

Figures shown here were produced using functions in the Seurat, Monocle and Clustree packages. Additional plots and customisations were created using the ggplot2 (v3.0.0) [30] and cowplot (v0.9.3) [31] packages. The analysis project was managed using the workflowr (v1.1.1) (50) package which was also used to produce the publicly available website displaying the analysis code, results and output.

## Results

Segmented epithelial nephrons, stroma and endothelial cells have been identified within human kidney organoids by correlating tissue morphology with established markers of equivalent cell types in mouse. However, several markers unique to a cell type within a developing kidney are also expressed in divergent cell types throughout the embryo. Therefore, it is unclear how closely kidney organoid cell types align with cells specified in an in vivo environment and characterising cell types derived from pluripotent cells in vitro must rely on combinations of markers unique to the cell type of interest. We employed scRNA-seq to analyse the cellular composition of kidney organoids, aiming to obtain profiles of the constituent cell types to complement and extend our prior characterisation by immunofluorescence.

### Defining the cellular composition of human kidney organoids using high-throughput single-cell RNA sequencing

**Fig. 1:**
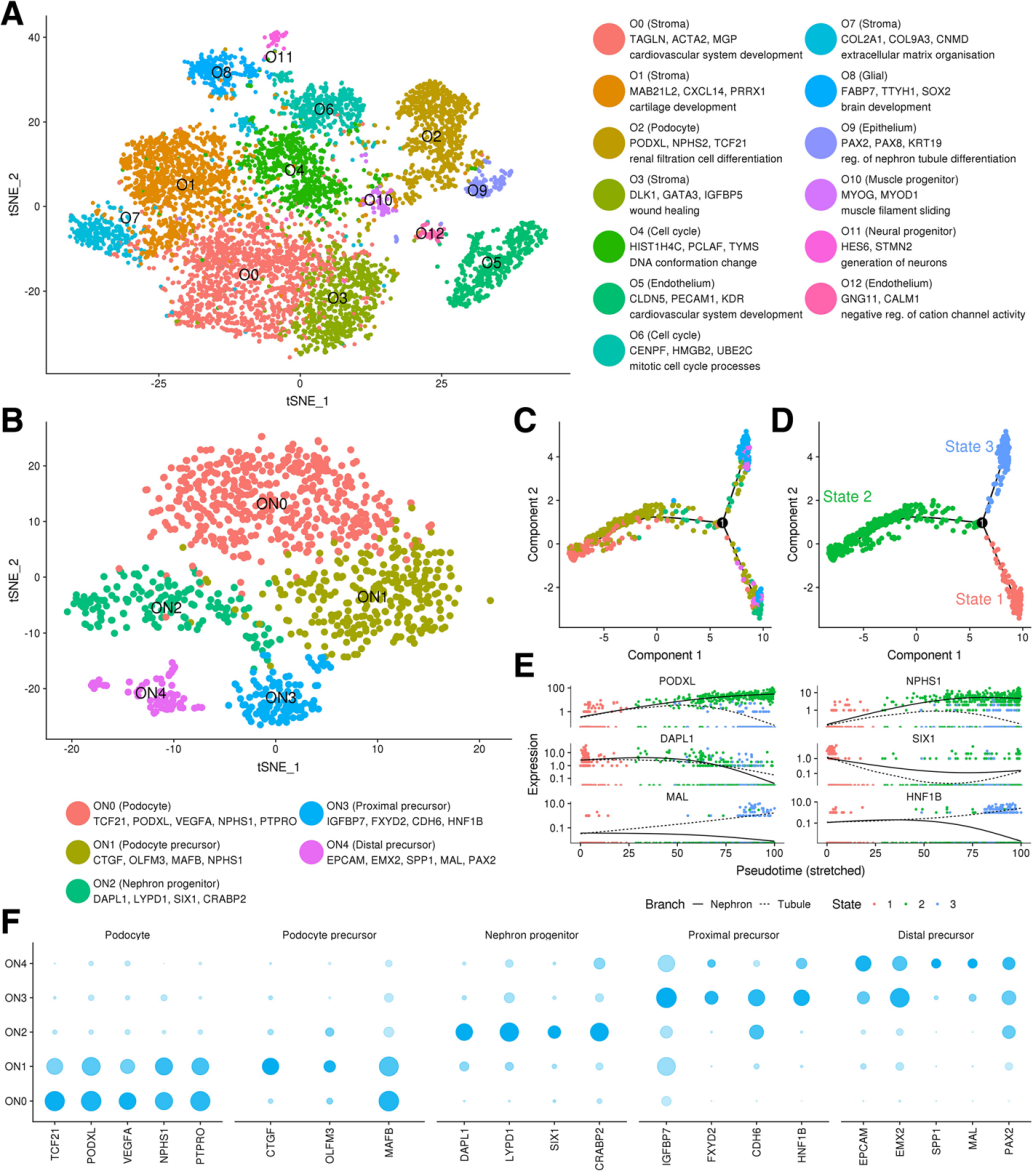
Single-cell RNA-seq profiling of human kidney organoids reveals expected and off-target populations. **a** tSNE plot revealing 13 distinct clusters (cluster O0 to cluster O12) identified from largest to smallest population as labelled. Clusters depicted in this figure have been referred to as organoid (O) followed by cluster number. Cluster identity indicated in colour key which includes select marker genes and highest ranking GO term for top 30 genes with positive log fold change values in each cluster. **b** Re-clustering of organoid nephron lineage cells from clusters O2 and O9 in **a** results in five nephron sub clusters as labelled. Cluster labels followed by marker genes expressed within the cluster. Clusters from this analysis have been referred to as organoid nephron (ON) followed by the cluster number. **c**, **d** Pseudotime trajectory analysis of organoid nephron cells supports a progression from nephron progenitor to podocyte, and proximal and distal nephron end points on different branches. Plot in **c** coloured by cluster identity in **b**. Plot in D coloured by Monocle state. **e** Expression of representative podocyte, nephron progenitor and tubular marker genes across the pseudotime trajectory coloured by Monocle state. **f** Dot plot representing key cell type marker gene expression within organoid nephron clusters. Dot size indicates proportion of cells in cluster expressing a gene, shading indicates the relative level of expression (low to high reflected as light to dark)

Our analysis of the sources of variation between kidney organoid differentiations included scRNA-seq data from over 8000 cells isolated from day 25 kidney organoids, identifying populations with similarity to nephron progenitor, nephron tubule, podocyte, endothelium and three stromal populations, as well as a small immune-like cluster and off-target neural populations [9]. That study highlighted experimental batch as the strongest contributor to transcriptional variation with evidence for shifts in relative maturation as a major confounder. In this study, we perform an independent analysis of that data (“Methods”). Each aspect of the current analysis is documented in a website at http://oshlacklab.com/combes-organoid-paper/, including further information and figures detailing quality control, clustering, marker and differential expression analysis [32]. We generated a combined dataset representing 7937 cells by integrating cells from four organoids across two batches (Additional file 1) and performed clustering over a range of resolutions (0–1 in 0.1 increments) using Seurat [20]. The Clustree package [26] was used to visualise how clusters changed with increasing resolution and select an appropriate resolution to use (Additional file 1). Thirteen organoid (O) clusters were identified at resolution 0.6 including clusters representing stromal, nephron, endothelial cells and cell types not usually present in a developing mouse kidney (Fig. 1a). Cluster identity was established by comparison of cluster marker genes to established markers of known murine and human renal cell types [10, 33–35] and by gene ontology (GO) analysis through ToppFun [36] (Additional file 2). The number and identity of clusters was largely consistent with our previous independent analysis [9], and the readily identifiable renal populations are dealt with below. Less identifiable and potentially off-target populations included a small neural population marked by *ELAVL3* and *ELAVL4*, and a more substantial population expressing glial markers including *FABP7*. An additional off-target population of muscle progenitors expressing *MYOD1*, *MYOG* and *PITX2* was detected for the first time (Fig. 1a). While smooth muscle cells play an important role in expelling renal filtrate from the kidney to the bladder, the presence of cells expressing muscle progenitor markers might suggest a developmentally off-target population. Immunofluorescence for MYOG identified scattered muscle progenitors in small numbers on the surface of the organoid (Additional file 1). FABP7-expressing presumptive glial cells were also present at a low frequency in the organoid stroma (Additional file 1). The presence of a detectable cluster for what appeared to be a relatively rare and peripheral cell population may result from differential dissociation of these populations during preparation of material for single-cell analysis. We were unable to detect cells expressing neural markers ELAVL3, ELAVL4 or NCAM1 with antibodies to these proteins. This neural cluster corresponds to a small proportion of the overall cells (0.9%). A lack of detectable cells within organoids when using antibody markers may reflect the rarity of the population or variations in the presence of this population between organoid differentiations.

The developing kidney is known to have distinct stromal subpopulations. While four stromal populations were evident within kidney organoids, unifying markers of these populations (e.g. *MEIS1*, *PDGFRA*, *COL3A1*) are expressed in stromal tissues throughout the mouse embryo and hence are not sufficient to identify these clusters as cell types specific to the developing kidney. One of these clusters (O3) is marked by GATA3 and is evident by immunofluorescence (Additional file 1). GATA3 is known to be expressed in mesangial cells within the glomerulus and in vascular associated cells [37]. In organoids, we observe expression of GATA3 in cells scattered throughout the stromal compartment.

### Analysis of organoid nephron cell types reveals a persistent nephron progenitor-like population

To interrogate the nephron and epithelial populations further, we re-clustered cells from clusters O2 (podocyte) and O9 (nephron epithelium) at multiple resolutions. We selected resolution 0.5 for analysis as this was the first resolution to split the epithelial cluster (O9) into subpopulations [32]. This re-clustering produced five organoid nephron (ON) clusters with similarity to nephron progenitor cells (ON2 marked by *DAPL1*, *LYPD1*, *SIX1*, *CRABP2*), podocyte precursors as seen in the proximal early nephron (ON1 *CTGF*, *OLFM3*, *MAFB*, *NPHS1*, low levels of *LHX1* and *PAX8*), podocytes (ON0 marked by *TCF21*, *PODXL*, *VEGFA*, *WT1*), proximal tubule precursors (ON3 *IGFBP7*, *FXYD2*, *CDH6*, *HNF1B*), and cluster expressing markers common to the ureteric epithelium and distal nephron (*EPCAM*, *EMX2*, *SPP1*, *MAL* and *PAX2* (ON4)) (Fig. 1b, Additional file 3). Though all of these cell types expressed markers conserved in mice, the top differentially expressed markers of nephron progenitor and proximal early nephron cells were recently defined markers specific to human kidney cell types [38, 39]. The *EPCAM*^+^ *PAX2*^+^ cluster ON4 also expressed markers enriched in the distal tubule such as *DEFB1* and *TMEM52B*. While we could find no markers previously identified as unique to the mouse ureteric epithelium, immunofluorescence for CDH1^+^GATA3^+^ structures previously defined as presumptive ureteric epithelium revealed the presence of these structures at a low frequency in the organoids profiled for scRNA-seq (Additional file 1). The reduced presence of this presumptive ureteric epithelium likely reflects the use of a ‘posteriorised’ differentiation using an initial culture (4 days of 8 μM CHIR). As previously described [3], this substantially reduces the presence of this epithelial segment. In addition, this cell type may be selectively lost due to inefficient dissociation. Some markers of maturing proximal (*SLC3A1*, *CUBN*) and distal tubule/loop of Henle (*UMOD*) were not detected in this data, despite being identified in previous bulk transcriptional profiling and immunofluorescence of older organoids (day 25) [40, 41]. Again, this may represent relative depletion of epithelial cell types during dissociation.

While our current kidney organoid protocol shows evidence of nephron formation and segmentation, previous bulk RNA-seq analysis suggested a peak of nephron progenitor marker expression early (days 14 to 17) followed by a significant decline with time [3]. We identify a cluster expressing nephron progenitor markers in these late (day 25) human kidney organoids (ON2). In the developing mouse kidney, nephron progenitors are thought to be dependent on supporting signals from the neighbouring ureteric tip and surrounding stroma, cumulatively referred to as the nephrogenic niche. This data suggests a nephron progenitor-like population is maintained in these kidney organoids apparently independent of the ureteric tip. Organoid stromal populations may be providing some supporting signals for these cells that likely represent progenitors that have failed to form a nephron.

We next used Monocle [28, 29] to order these organoid nephron cells along a continuous pseudotime trajectory and visualised cell types by cluster (Fig. 1c) and by Monocle-determined cell state (Fig. 1d). Based on mouse lineage tracing [42] and single-cell trajectories in human [38], we would expect a nephron progenitor state to diverge into separate trajectories for podocyte, distal and proximal tubule. This expected trajectory is maintained in the organoid cell types with a nephron progenitor-enriched population that diverges into separate trajectories for podocyte and nephron tubule cell types. The proximal early nephron cluster (ON1) leads to a more mature podocyte endpoint (ON0) as predicted from the marker analysis. The proximal and distal nephron precursor clusters occupy a separate trajectory, analogous to similar results in the developing human kidney [38]. Gene expression along the pseudotime trajectory and across cell clusters shows expected segregation of key cell type markers (Fig. 1e, f). This analysis suggests that organoid nephron differentiation proceeds in a manner analogous to that which occurs in an in vivo environment.

Overall, this analysis of kidney organoid scRNA-seq data supports the presence of previously characterised nephron and endothelial cell populations while increasing confidence in each by identifying robust sets of co-expressed cell type-specific markers. New insight revealed by this approach includes identification of a persistent nephron progenitor-like population, stromal subpopulations, and off-target glial, neural and muscle progenitor populations.

### Comparison to human developing kidney reveals conserved endothelial, nephron and stromal populations

**Fig. 2:**
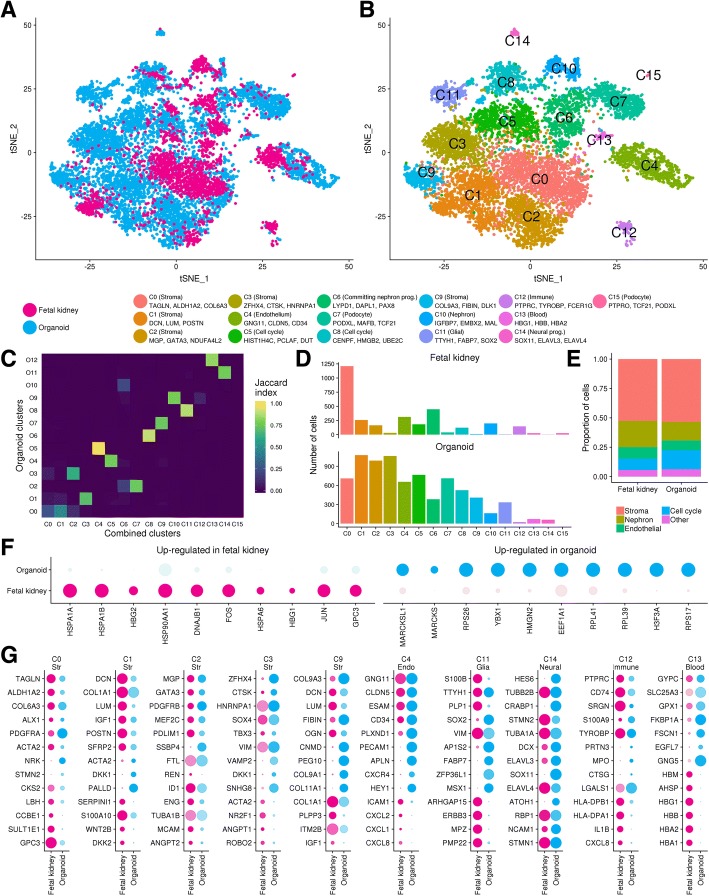
Integration and comparison of kidney organoids and human fetal kidney scRNA-seq. **a** tSNE plot of combined organoid and hFK data coloured by sample type. **b** tSNE plot revealing 16 ‘combined’ (C) clusters identified from largest to smallest population (C0–C15). Cluster identity and select conserved marker genes shown next to cluster colour key. **c** Comparison of organoid cell clustering in ‘organoid only’ to ‘combined’ clusters. Overlap in samples between clusters from the different analyses is shown using the Jaccard Index with a score of 1 (yellow) indicating identical clusters and 0 (blue) indicating no cells in common. **d** Number of cells contributing to each cluster from hFK and organoid samples. **e** Comparison of general cell type composition between organoid and hFK samples. Stroma includes C0, C1, C2, C3, and C9; nephron includes C6, C7, and C10. **f** Differentially expressed genes with largest fold changes between all organoid and all hFK cells. **g** Top conserved markers and differentially expressed genes between datasets for clusters from the ‘combined’ analysis. Cell cycle clusters not displayed. Similar analysis for nephron clusters is presented in Fig. 3

Recent transcriptional comparisons between kidney development in mouse and human have identified human-specific cell type markers [10, 39], many of which were expressed in the human kidney organoid nephron cell types detailed above. Despite expression of these key markers, the possibility of broader underlying differences between nephron cell types in kidney organoids and the developing human kidney remained. Likewise, the extent of similarity between organoid and human fetal kidney stromal cell types was unclear. To facilitate direct comparison between organoid and human fetal kidneys, we combined our organoid single cell data with published hFK single cell data [10] using the Seurat alignment method based on canonical correlation analysis and dynamic time warping [20]. The hFK cells were obtained at 16 weeks of development, a period of active branching morphogenesis and nephron formation [10]. The combined dataset represented expression information for 18,812 genes within 11,115 cells. We again clustered at multiple resolutions and used a clustering tree to select a resolution of 0.5 for analysis. This resulted in 16 combined (C) clusters including five stromal clusters (C0, C1, C2, C3, C9), an endothelial cluster (C4), four nephron lineage clusters (C6, C7, C10, C15), two clusters related to cell cycle (C5, C8), glial (C11) and neural (C14) clusters, an immune cluster (C12), and a blood cell cluster (C13) (Fig. 2a, b; Additional file 4). Organoid cells were grouped in similar clusters in the combined data set compared to the previous organoid-only analysis (Fig. 2c) suggesting that we have identified the same populations in both the organoid-only and combined analyses.

We anticipated differences in the cell types present in fetal kidney and kidney organoids as the hFK data represented cells dissociated from the nephrogenic zone of the outer cortex rather than the whole organ. Previous analysis showed the hFK dataset contains cortical stroma, early nephron, vascular, blood and immune cell types, but it does not contain ureteric epithelium, mature nephrons or medullary stromal populations [10]. Conversely, kidney organoids contain populations designated as off-target given they were not intentional outcomes of the kidney organoid differentiation protocol. With these caveats in mind, we assessed the similarity between cell types in these datasets by determining the contributions of kidney organoid and hFK cells to each cluster (Fig. 2d), and the proportions of general cell types between the samples (Fig. 2e). This showed substantial contributions of both organoid and hFK cells to stromal, endothelial and nephron clusters. Importantly, this combined analysis allowed us to allocate small numbers of cells to clusters not previously identified in the individual tissue analysis such as a small group of organoid cells expressing immune response genes within C12, and fetal kidney cells expressing glial and neural markers within C11 and C14. Neural cells were not identified in the original analysis of this hFK data [10] but these cells have been found at low frequency in other datasets from the human fetal kidney [43].

### Top tissue type differences relate to cellular stress in the fetal kidney data and growth in organoids

Co-clustering the organoid and hFK data provided a means to analyse conserved and differential transcriptional profiles within cell types. Initial differential expression analysis between hFK and organoid cell types within each cluster revealed recurring sets of genes that confounded further analysis. We performed differential expression analysis between all cells in the organoid and hFK samples to formally identify genes that were enriched in either dataset (Fig. 2f). GO analysis of genes upregulated in the hFK cells returned terms including ‘response to unfolded protein’, ‘regulation of programmed cell death’ and ‘response to temperature stimulus’, consistent with heat shock and cellular stress. GO terms associated with organoid enriched genes relate to an abundance of ribosomal proteins, including ‘translation initiation’ and ‘ribosome biogenesis’, consistent with enhanced growth (Additional file 5). This heat shock or stress signature in the hFK data is a potential side effect of cell dissociation [44] but could also reflect the fact that the fetal material was acquired at termination and may have been subjected to suboptimal conditions prior to processing. We note this signature is only revealed after comparing to another dataset and is a potential drawback with using cells derived from primary tissue, which is otherwise an ideal reference. The ribosomal RNA signature enriched in kidney organoids may reflect a higher metabolic rate within kidney organoid cells in culture [45]. Most tissue culture media, including that used for kidney organoid culture, is high in glucose with culture conditions also physiologically hyperoxic compared to the fetus.

### Conserved and differential expression analysis between fetal and organoid cell types

The major differences between the hFK and organoid datasets are likely to be technical artefacts due to sample isolation and culture conditions. To focus on biological differences between cell types, we removed the genes identified as sample-specific from subsequent differential expression analyses between the datasets. We then compared hFK and organoid cells within each cluster to assess conserved and differentially expressed genes (Additional files 4 and 6), summarised in Table 1 and Fig. 1g.

**Table 1:** Summary of gene expression analyses for combined clusters excluding cell cycle and nephron lineage clusters

Cluster and sample origin		DE	Top enriched genes	GO summary for ≥ 10 DE genes	Top cluster markers	Top conserved markers
C0 Stroma	hFK	3	CKS2, LBH, CCBE1		SULT1E1, GPC3, MEG3, SERPINH1, ALDH1A2, TAGLN	TAGLN, ALDH1A2, PDGFRA, ZEB2, ACTA2, ALX1, SNAI2, COL6A3
	Org	2	NRK, STMN2			
C1 Stroma	hFK	87	SERPINI1, COL1A1, S100A10, SFRP2, ANXA1, ASPN	ECM organisation, signalling receptor binding	DCN, COL1A1, LUM, IGF1, POSTN, SFRP2, COL1A2, OGN	IGF1, SFRP2, COL1A1, DCN, LUM, COL1A2, COL3A1, POSTN
	Org	5	RPL27A, PALLD, ACTA2, DKK1			
C2 Stroma	hFK	11	REN, ID1, PDLIM1, ITGA8, ENG, CPM	Endothelial and smooth muscle development	DLK1, MGP, NDUFA4L2, GATA3, APOE, PDGFRB, MEF2C	MGP, GATA3, NDUFA4L2, MEF2C, ACTA2, PDGFRB, PDLIM1
	Org	2	SSBP4, FTL			
C3 Stroma	hFK	23	ACTA2, NR2F1, ANGPT1, ROBO2	No significant results	MAB21L2, CXCL14, PRRX1, ZFHX4, MAB21L1, CD24, COL9A2	ZFHX4, CTSK, HNRNPA1, DNM3OS, SOX4, LIMA1, TBX3
	Org	12	VIM, VAMP2, SNHG8, DKK1	Mitochondrial ribosome binding		
C4 Endothelium	hFK	45	FN1, RBP5, PLPP3, CCL21, LGALS1, CXCL1, CXCL2, CXCL8, ICAM1	Signalling receptor binding, chemokine activity	GNG11, EGFL7, CLDN5, ESAM, PVLAP, CD34, CAV1, ARHGAP29, APLN	GNG11, EGFL7, CLDN5, ESAM, PVLAP, S100A16, ARHGAP29, APLNR, CAV1, CD34, KDR, TIE1
	Org	44	APLN, PECAM1, MMP1, CAV1, CXCR4, HEY1	Angiogenesis, vascular development		
C9 Stroma	hFK	67	COL1A1, PLPP3, ITM2B, IGF1, SPON2	Cell adhesion, ECM organisation	COL2A1, COL9A3, CNMD, MIA, COL9A2, COL9A1, FIBIN	COL9A3, DCN, LUM, FIBIN, COL1A2, OGN, IGFBP6, COL1A1, SOX9, SFRP2, MGP
	Org	41	CNMD, PEG10, COL9A1, COL9A3, GNG5, COL11A1	ECM organisation, cartilage dev.		
C11 Glia	hFK	203	S100B, PLP1, MPZ, PMP22, ARHGAP15	Axon development	AP1S2, TTHY1, FABP7, SOX2, MSX1, PCSK1N	S100B, GPM6B, TTYH1, PLP1, SOX2, NKAIN3, PMP22, CNP, VIM
	Org	136	AP1S2, FABP7, ZFP36L1, MSX1	ATP synthesis, cell respiration		
C12 Immune	hFK	88	HLA-DPB1, HLA-DPA1, HLA-DRB1, CXCR4, CD83	Response to IFN gamma, antigen binding	HLA-DRA, CD74, SRGN, S100A9, TYROBP, S100A8, HLA-DPB1, LYZ	SRGN, LYZ, S100A9, TYROBP, S100A8, FCER1G, SPP1, FTL, CD74
	Org	24	PRTN3, MPO, CTSG, AZU1	Immune response, defence response		
C13 Blood	hFK	24	HBM, AHSP, ALAS2, HEMGN, SLC25A37	Erythrocyte development, oxygen transport	HBG1, HBB, HBA2, HBA1, HMB, AHSP, ALAS2, SCNA, GNG11, HEMGN	CYPC, SLC25A39, SLC25A37, GPX1, HEY1, COPZ1, PRDX2, ACTB, SELENBP1, PFN1
	Org	168	FSCN1, EGFL7, FKBP1A, EIF4G2, GNG5, TPM4, BAX	Viral process, translation initiation		
C14 Neural	hFK	N/A			HES6, CRABP1, TUBB2B, STMN2, TAGLN3, SSTR2	N/A
	Org	N/A				

Abbreviations: *DE* differentially expressed (adjusted *p* value < 0.05, absolute log fold change greater than 0.8), *ECM* extracellular matrix. Full lists available in Additional files and on website [32]

### Conserved stromal and endothelial cell types between organoid and fetal kidney

Our understanding of stromal subpopulations in developing human kidneys is relatively poor. However, Lindstrom and colleagues sampled the cortex of the developing human kidney and classified the stromal cells within this sample into five populations representing three major groups: Lindstrom cluster (L) 9, marked by *REN*, *MGP* and *GATA3*; L2 marked by *LUM*, *SFRP2* and *DCN*; and three grouped subpopulations L10–12 with markers including *TCF21*, *ALDH1A2*, *ANGPT1*, *MEIS1*, and *TAGLN* [10]. These populations appear to be conserved in kidney organoids with the combined cluster C0 correlating to the L10–12 clusters (conserved markers include *TCF21*, *ALDH1A2*, *ANGPT1* and *TAGLN*); C1 representing L2 (conserved markers include *LUM*, *SFRP2*, and *DCN*); and C2 representing L9 (conserved markers include *MGP* and *GATA3*). Thus, human kidney organoids contain stromal populations similar to hFK cortical stromal populations, which form part of the nephrogenic niche and have been shown to influence nephron formation in mice [46, 47]. Few genes are differentially expressed between hFK and organoid cells in C0 but these include *NRK* and *STMN2*, upregulated in organoid cells, and *CCBE1* and *LBH* upregulated in hFK. More substantial differences are apparent in C1 with 87 genes upregulated in hFK compared to organoid cells including several signalling molecules such as *SFRP2*, *IGF1*, *WNT2B*, *DKK2* and *SEMA3A*. The expression of *WNT2B* is notable as this ligand is not expressed in the developing mouse kidney, and WNT signalling has several critical roles in kidney development. hFK cells within Cluster C3 express higher levels of markers associated with vascular smooth muscle and mesangial development including *RENIN* [48]. However, several highly upregulated markers are conserved within this cluster and the conserved signature also features several smooth muscle-associated genes such as *GATA3*, *MEF2C*, *ACTA2*, *HOPX*, *ANGPT2* and *PDGFRB*. Stromal clusters C3 (marked by *MAB21L2*, *CXCL14*, *PRRX1*) and C9 (*COL2A1*, *COL9A3*, *CNMD)* had smaller contributions of hFK cells (less than 5% of total cells), which could indicate that these organoid stromal clusters are less similar to native cell types, or that equivalent stromal populations are not adequately represented in the hFK sample. In situ hybridisation results from the mouse embryo indicate that some of the most upregulated markers of organoid stromal cell types C3 and C9 are expressed in the medullary and ureteric stroma of the developing kidney (Additional file 1), which was not sampled in the hFK data.

The endothelial cluster C4 featured extensive conservation of established markers such as *CLDN5*, *CDH5*, *CD34*, *KDR*, *TIE1*, *SOX17*, *SOX7* and *FLT1*. GO analysis of conserved markers resulted in terms related to vascular development indicating congruence between organoid endothelial cells and those from the hFK data. Despite this conservation, several genes were enriched in hFK or organoid endothelial cells. hFK upregulated genes were associated with cell signalling including chemokines *CXCL1*, *CXCL2* and *CXCL8* whereas organoid cells expressed higher levels of endothelial markers such as *PLXND1*, *APLN* and *PECAM1*. As such organoid endothelial cells appear to be appropriately specified but differ in the complement of signalling molecules they express. Whether this is the result of cell intrinsic factors or a response to being embedded in a different stromal environment is unclear.

### Glial, neural, immune and blood clusters

Some clusters in the combined analysis consisted mainly of cells from one dataset. Organoid-enriched clusters include a glial cluster C11 (marked by *TTYH1*, *FABP7*, *SOX2*), which included less than ten hFK cells, and C14 (marked by *SOX11*, *ELAVL3*, *ELAVL4*) that mostly contained organoid cells. GO analysis of the top markers of these clusters identifies terms associated with glial cell differentiation (C11) and generation of neurons (C14). There is some evidence of neural precursors being present during mouse [49] and human [43] kidney development, and neural populations play an important role in adult renal physiology. However, neural and renal progenitors have distinct embryonic origins, the former from the ectoderm and the latter from the intermediate mesoderm [6, 50, 51]. As our organoid protocol directs the bulk of cells towards an intermediate mesoderm-like fate [3], these glial and neural cell types are considered off-target and may reflect cells that adopted an alternative identity during the early stages of differentiation and persisted in culture.

An immune cell cluster (C12, marked by *PTPRC*, *TYROBP*, *FCER1G*) was mostly derived from hFK cells but surprisingly, 20 organoid cells contributed to this cluster and shared expression of immune response genes. During development, haematopoietic progenitors, cells can arise from specialised endothelial cells termed hemogenic endothelium [52]. This type of endothelium occurs within the aorta-gonad-mesonephros region [53], which is adjacent to the site of kidney development. Markers of hemogenic endothelial cells such as *PECAM1*, *KDR*, *KIT* and *CDH5* [52] are expressed in the organoid vasculature and, as such, it is possible that kidney organoids have some capacity to generate cells involved in the immune response.

Top markers of cluster C13 included genes highly expressed in blood (*HBG1*, *HBB*, *HBA2*), a signature primarily driven by the hFK-derived cells within the cluster as organoid-derived cells within this cluster did not share expression of these cell type-specific markers. GO analysis of upregulated organoid genes, and genes that were conserved markers between fetal and organoid cells in this cluster led to terms that were not related to blood.

### Conserved nephron progenitor and early nephron cell types

hFK and organoid cells were present in clusters representing nephron progenitor cells (C6) and nephron epithelium (C10), but other hFK cells were split between a cluster containing most organoid podocytes (C7) and a hFK-specific podocyte cluster (C15).

### Congruence and differences between hFK and kidney organoid nephron cell types

**Fig. 3:**
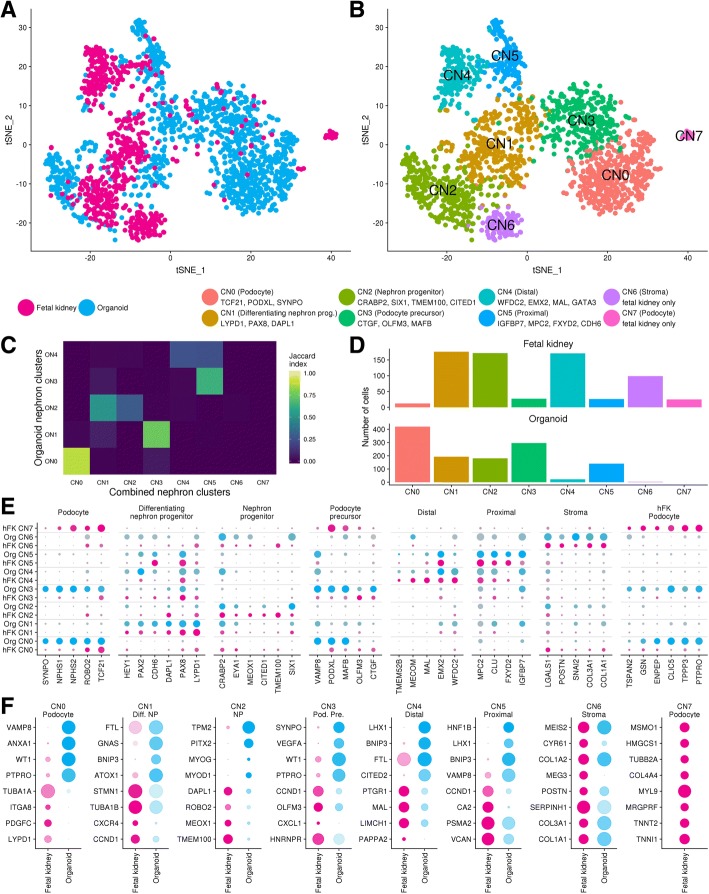
Comparison of nephron cell types within kidney organoids and human fetal kidney. **a**, **b** Sample of origin and re-clustering of combined nephron (CN) lineage cells results in eight clusters. Cluster identity and select conserved marker genes shown next to cluster colour key. Cells for this analysis were selected from combined clusters C6, C7, C10 and C15. **c** Comparison of organoid cells between organoid nephron (ON) and combined nephron (CN) clusters. Colours show overlap in cells between clusters according to the Jaccard Index. **d** Number of cells in each combined nephron cluster by dataset. **e** Split dot plot showing relative expression for select marker genes within organoid and hFK cells in the combined nephron clusters. hFK data in pink, organoid in blue. Circle size represents the proportion of cells in the cluster expressing that gene, shading indicates expression level (low to high reflected as light to dark). **f** Top differentially expressed genes between datasets within combined nephron clusters. Chart colouring and shading as per **e**. Results for CN6 and CN7 are not differential expression results as few (CN6) or no (CN7) organoid cells are present within these clusters. These instead reflect top cluster markers (CN6) or markers enriched in CN7 but not CN0 or CN3. Organoid expression values for CN6 are derived from three organoid cells within this cluster

To compare nephron subpopulations in detail, we re-clustered cells from the combined nephron lineage clusters (C6, C7, C10, C15) in isolation. This generated seven combined nephron (CN) clusters with cells from hFK and organoid co-clustering within populations representing nephron progenitors (CN2), differentiating nephron progenitors (CN1), distal (CN4) and proximal (CN5) nephron segments, podocyte precursor (CN3) and podocyte cells (CN0). The hFK-specific podocyte cluster (CN7) noted previously was maintained in this analysis, and a new cluster of hFK stromal cells, marked by *COL3A1*, *POSTN* and *MEG3* (CN6) was resolved. CN6 does not express any known nephron markers aside from *TMEM100*, which was reported to be specific to nephron progenitors in the human fetal kidney [10]. In the absence of other nephron progenitor markers, this cluster does not appear to be part of the nephron lineage (Fig. 3a, b). Cells within this stromal CN6 cluster may have been associated with the nephron lineage based on the broad expression of stromal markers within human nephron progenitor cells [10]. Again, organoid nephron cells formed similar groups when clustered with the hFK data as when clustered alone (Fig. 3c). hFK and organoid cells contributed to most clusters with the largest contributions in the nephron progenitor and differentiating progenitor cluster (Fig. 3d). hFK cells were scattered through the two organoid podocyte clusters but again a group of hFK podocyte cells remained separate. In the clusters representing the nephron epithelium, organoid cells were located in the proximal nephron cluster with few cells contributing to the distal while the hFK cells displayed the opposite pattern, consistent with the previous hFK analysis [10] and the under representation of distal nephron in these organoid samples.

Key cell-type markers were conserved between the datasets but differential expression testing revealed underlying differences in transcriptional profiles between hFK and organoid nephron cell types (Table 2, Fig. 3e, f, Additional files 7 and 8). hFK cells within nephron progenitor clusters showed enhanced expression of progenitor genes *TMEM100*, *MEOX1*, *ROBO2* and *DAPL1*, and a small portion of organoid nephron progenitors expressed low levels of muscle progenitor genes *MYOD1* and *MYOG* (Additional file 1). Thus, despite the conservation of key markers, this population may be adopting a muscle progenitor fate in the absence of appropriate signals to reinforce nephron progenitor identity. Alternatively, a small number of muscle progenitors may have clustered with the nephron progenitors due to similarities in their expression profiles. Likewise, underlying a conserved profile, hFK cells within the differentiating nephron progenitor cluster expressed elevated levels of proliferation associated genes such as *CCND1* and *PCLAF* likely reflecting higher proliferation rates as seen during commitment to nephron formation in vivo [54].

**Table 2:** Summary of gene expression analyses for combined nephron clusters excluding stromal cluster CN6

Cluster and sample origin		DE	Top enriched genes	GO summary for ≥ 10 DE genes	Top cluster markers	Top conserved markers
CN0 Pod	hFK	79	TUB1A1, NR2F1, SOX4, LGALS1, NUSAP1, LYPD1	Central nervous system development	S100A6, TCF21, PODXL, TPPP3, SBSPON, MMP5, CPXM1, DUSP23, THSD7A, MME	TCF21, MME, PODXL, THSD7A, TPPP3, SBSPON, MAFB, ENPEP, ROBO2, NPHS2
	Org	175	VAMP8, ANXA1, AIF1, S100A4, TGFBR3, WT1	ATP synthesis, oxidative phosphorylation		
CN1 Diff. NP	hFK	37	CCND1, PCNA, CXCR4, TUBA1A, TBUA1B, STMN1	mRNA splicing	LYPD1, PAX8, HIST1H4C, PCLAF, CDH6, DAPL1, CCND1, RBP1, HMGB2	LYPD1, PAX8, HIST1H4C, PCLAF, DAPL1, PCP4, HEY1, CDH6, RBP1, PAX2
	Org	4	FTL, GNAS, BNIP, ATOX1			
CN2 NP	hFK	24	TMEM100, ITM2C, MEOX1, EPCAM, ROBO2, DAPL1	Amyloid precursor biosynthesis	ACTC1, NNAT, MYLPF, MYL1, TMEM100, TPM2, CRABP2, TUB1A1	NNAT, CRABP2, TUB1A1, IGF2, SIX1, TMEM100, CITED1, SOX4, MEOX1, MEIS2
	Org	25	ACT1C1, MYLPF, TPM2, PITX2, MYOG, MYOD	Muscle filament sliding		
CN3 Pod. Pre.	hFK	37	CCND1, OLFM3, CXCL1, HNRNPR, STMN1	No significant BP terms	CTGF, GPX3, TSPAN8, PAPPA, PAPPA, ITIH5, SERINC5, HES4, NPHS2, NPHS1, MAFB, PTPRO	CTGF, OLFM3, BCAM, NPHS1, ARHGAP29, CLDN1, LEPROT, TMP1, STON2, CLDN5, MAFB
	Org	78	GPX3, ANXA1, S100A4, AIF1, PTPRO, SYNPO, VEGFA, WT1	Epithelial cell diff. involved in kidney development		
CN4 Distal	hFK	26	PTGR1, MAL, LIMCH1, ELF3, ALDH1A1, PAPPA2	Epithelium development	HBG2, ATF3, LIMCH1, MAL, WFDC2, BTG2, HES1, KLF6, MECOM, ELF3, TUBB2B, GATA3	WFDC2, EMX2, LIMCH1, MAL, TUBB2B, SAT1, MECOM, HMGA1, HES1, GATA3, ATP1B1, GNG11
	Org	11	LHX1, BNIP3, FTL, CKB, BASP1, CITED2, HNRNPAB	Kidney morphogenesis		
CN5 Proximal	hFK	34	CCND1, CA2, VCAN, ELF3, DCDC2, FLRT3	No significant BP terms	IGFBP7, CD24, PCP4, PCSK1N, FXYD2, EMX2, MPC2, APOE, CLU, CFAP126, FTL, ATP1B1	IGFBP7, MPC2, SMIM24, FLRT3, EMX2, FXYD2, GNG11, TSPAN12, CLU, PCP4, ATP1B1, PDZK1
	Org	14	PCSK1N, CITED2, BNIP3, S100A13, MLLT1, PRDX5, VAMP8, LHX1	Ureteric bud morphogenesis, pronephros development		
CN7 hFK Pod	hFK				CXCL12, TNNI1, TNNT2, MME, MYL9, MRGPRF, TPPP3, ANXA2, COL4A4, ADM, PTPRO, MSMO1	N/A
	Org					

Abbreviations: *DE* differentially expressed (adjusted *p* value < 0.05, absolute log fold change greater than 0.8), *hFK* human fetal kidney, *Org* organoid. GO summary reporting top significant Gene Ontology (GO) Biological process (BP) results when fetal kidney or organoid DE gene lists were greater than or equal to ten genes. Full lists of cluster markers, conserved and differentially expressed genes and corresponding GO analyses available in Additional files or on website [32]

Most of the top markers of the nephron tubule clusters represented established markers of distal and proximal tubule and were conserved between hFK and organoid cells. hFK cells expressed higher levels of distal markers *MAL*, *LIMCH1* and *PAPPA2* in distal cluster CN4 and organoid cells in proximal cluster CN5 had increased expression of genes associated with ureteric bud and pronephros development.

Of the three podocyte clusters, CN3 featured conserved expression of recently defined human podocyte precursor markers *MAFB*, *CTGF* and *OLFM3* [38] though *OLFM3* was expressed at higher levels in hFK cells in this cluster and organoid cells had higher levels of several podocyte markers such as *PTPRO*, *SYNPO*, *VEGFA* and *WT1*. hFK and organoid cells in CN0 expressed podocyte markers *TCF21*, *POXDL*, *ROBO2* and *NPHS2* but hFK cells within this cluster maintained expression of human nephron progenitor markers *LGALS1* and *LYPD1* [38] suggesting these cells may represent a more progenitor-like state than the organoid cells in this cluster. A final hFK-specific podocyte cluster formed distinct from the other two clusters which still included podocyte markers such as *PTPRO* and *TCF21* as marker genes but also included genes such as *TNNI1*, *TNNT2* and *MYL9*, which are expressed in cardiac muscle and podocytes. As markers of CN0 and CN7 largely overlap and represent a maturing podocyte state (*POXDL*, *PTPRO*, *TCF21 AIF1*), we investigated disparities between these clusters by performing differential expression analysis (Additional file 9).

Podocyte genes *COL4A4* and *ANXA2* as well as *TNNI1*, *TUBA1A*, *COL9A1*, *STMN1* and *CA2* were upregulated in hFK-specific CN7. Genes upregulated in CN0 included podocyte enriched genes *ANXA1*, *GPX3*, *VAMP8*, *DACH1* and *WT1* as well as additional genes related to ATP synthesis that likely relate to the increased growth rate in culture. While CN0 expresses established podocyte markers, this cluster may also contain podocyte precursor states or podocytes that have not yet matured into glomeruli. Such a distinction may underlie the separation between CN0 and CN7.

This comparative analysis of nephron cell types within kidney organoids and human fetal kidney shows strong conservation of key cell type-specific markers while uncovering differences in the expression levels of key nephron progenitor markers, and a separation of some hFK podocytes from others, potentially reflecting in vivo maturation. We did not observe differences in transporter expression or markers of tubule maturation between organoid and hFK samples but that may be due to insufficient depth of profiling in these scRNA-seq datasets.

## Discussion

We performed an in-depth analysis of nephron subpopulations in kidney organoids and found co-expression of a robust suite of established cell type-specific markers. Pseudotime analysis of these cell types suggests organoid nephron formation replicates an expected developmental trajectory from nephron progenitor to podocyte and tubular end points. We then asked whether there were underlying differences between kidney organoid cell types and equivalent populations in the developing human kidney. Organoid and hFK single-cell RNA-seq datasets were integrated and clustered, with cells from both datasets contributing to most clusters. Conserved gene expression between organoid and hFK cells within endothelial, stromal and nephron cell types revealed congruence between these cell types demonstrating the capacity of organoids to represent many aspects of the developing human kidney. Where immunofluorescence had identified the presence of stromal markers in organoids, our single-cell analysis identified five stromal subpopulations, at least three of which are conserved to some level in the developing human kidney. The remaining two may represent renal stromal populations that are simply not represented in the hFK data set due to limited tissue collection, or off-target stromal cell types. Further comparisons with more complete human data sets will be required to discern between these options.

A recent study from Wu et al. also used single-cell analysis to analyse our kidney organoid protocol across time and compare it to another organoid protocol and human kidney cell types [55]. While the renal populations and congruence with human kidney cell types we identify are consistent with those results, the relative proportion and types of cells captured by that analysis differs somewhat from our findings. For example, we detect a muscle progenitor population, where they detected a cluster of melanocytes. We detect a glial and a neural cluster and they detected a neural progenitor population and four neuron clusters. The proportion of off-target populations in those samples is higher than in our analysis (~ 20% Wu et al., 6% this study), as is the proportion of tubule to podocyte cells. As such, the proportions of cell types generated by the same differentiation protocol are likely to vary between laboratories.

We were unable to resolve a distinct population representing ureteric epithelium in either dataset; however, markers previously used to define this population were under represented in the organoids analysed in this study. We previously reported simultaneous generation of presumptive ureteric epithelium and nephron lineages, with the proportions of cell types generated dependent on the timing of exposure to signals that pattern the anterior-posterior axis of the intermediate mesoderm [3]. The organoids generated for the present study were distinctly posterior and hence contained a lower frequency of epithelial GATA3^+^ structures. Recent studies argue that ureteric epithelium and nephron lineages cannot be generated simultaneously because they arise from distinct regions during embryonic development and instead must be generated using distinct protocols [7]. Analysis of this epithelial GATA3^+^ cluster from other kidney organoids is required to further explore the identity of this cell type. However, the absence of any ureteric epithelial cluster in the hFK data suggests a resistance of this tubular epithelium to dissociate into single cells. This same population may be resistant to single-cell isolation from organoids. Hence, this will need to be overcome, perhaps using alternative technologies such as nuclear RNA preparations.

Differential expression within combined organoid and fetal kidney clusters identified an upregulation of ribosomal genes in kidney organoids and a heat shock/unfolded protein response in the fetal kidney data, both likely the result of technical artefacts rather than fundamental differences in cell identity. Examining gene expression after excluding these sample-enriched genes revealed additional differences between hFK and organoid cell types including notable changes in the levels of expression of nephron progenitor marker genes and in the levels and repertoire of growth factors expressed by stromal and endothelial cells. What was not evident in either dataset was the expression of several cell type-specific ligands and receptors that are apparent in analogous single cell datasets from the developing mouse kidney [33, 56]. For example, genes such as *GDNF* and *RET*, which encode a key ligand and receptor pair are known to operate in human kidney development as both genes cause renal birth defects when mutated in humans [57]. Being unable to detect the expression of such important genes in the reference hFK dataset leaves the possibility of important differences between organoid and hFK cell types that may only be revealed with deeper profiling.

## Conclusions

This analysis supports a conservation of cell types between organoids and human fetal kidney. Overall, the data presented here builds confidence in the fidelity of organoid nephron, stromal and endothelial cell types, which will encourage disease modelling and drug screening efforts in human kidney organoids.

## Additional files

Additional file 1: Supporting analysis for organoid data set. Figure with quality control, integration and supporting analysis for organoid dataset. (PNG 7123 kb)

Additional file 2: Organoid cluster markers. Organoid cluster markers and associated GO terms. (XLSX 3062 kb)

Additional file 3: Organoid nephron cluster markers. Organoid nephron cluster markers and associated GO terms. (XLSX 1089 kb)

Additional file 4: Combined conserved markers. Markers conserved between organoid and hFK cells within combined clusters and associated GO terms. (XLSX 77 kb)

Additional file 5: Differential expression analysis between organoid and hFK samples. Results from differential expression analysis between organoid and hFK samples used to identify a signature representing the main differences between the organoid and hFK datasets. (XLSX 2050 kb)

Additional file 6: Differential expression analysis for combined clusters. Results from differential expression analysis for organoid and hFK cells within combined clusters. (XLSX 2893 kb)

Additional file 7: Combined nephron conserved markers. Combined nephron cluster markers and associated GO terms. (XLSX 673 kb)

Additional file 8: Differential expression analysis for combined nephron clusters. Results from differential expression testing between organoid and hFK cells within each combined nephron cluster after removal of the sample-enriched signature. (XLSX 2040 kb)

Additional file 9: Differential expression analysis between podocytes in CN0 vs CN7. Results for differential gene expression testing between hFK-specific podocyte cluster CN7 and mixed organoid and hFK podocyte cluster CN0. (XLSX 77 kb)
