# Supplementary figures and images for: Single-cell analysis reveals congruence between kidney organoids and human fetal kidney
Source: Genome Med. 2019 Jan 23;11:3. doi: 10.1186/s13073-019-0615-0 (PMC6345028; doi:10.1186/s13073-019-0615-0)

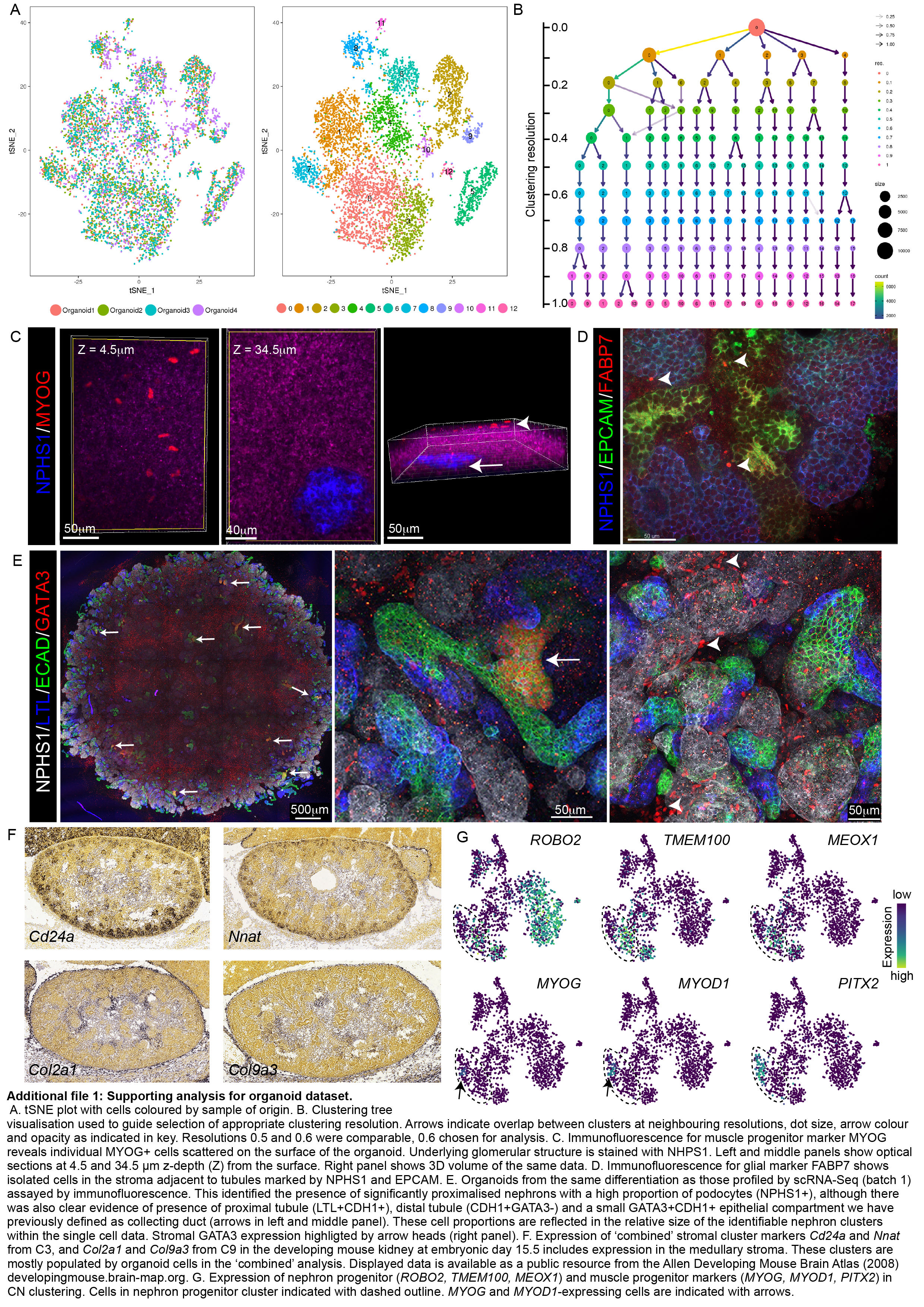

Supplement: Supplementary file 1 — Supporting analysis for organoid data set. Figure with quality control, integration and supporting analysis for organoid dataset. (PNG 7123 kb) [file 13073_2019_615_MOESM1_ESM.png]
